# Self-Explaining Social Robots: An Explainable Behavior Generation Architecture for Human-Robot Interaction

**DOI:** 10.3389/frai.2022.866920

**Published:** 2022-04-29

**Authors:** Sonja Stange, Teena Hassan, Florian Schröder, Jacqueline Konkol, Stefan Kopp

**Affiliations:** ^1^Social Cognitive Systems Group, Faculty of Technology, Bielefeld University, Bielefeld, Germany; ^2^Robotics Group, Faculty 3–Mathematics and Computer Science, University of Bremen, Bremen, Germany

**Keywords:** explainability, transparency, social robots, human-robot interaction (HRI), interaction architecture, autonomous explanation generation, user-centered explanation generation, socio-interactive explanation generation

## Abstract

In recent years, the ability of intelligent systems to be understood by developers and users has received growing attention. This holds in particular for social robots, which are supposed to act autonomously in the vicinity of human users and are known to raise peculiar, often unrealistic attributions and expectations. However, explainable models that, on the one hand, allow a robot to generate lively and autonomous behavior and, on the other, enable it to provide human-compatible explanations for this behavior are missing. In order to develop such a self-explaining autonomous social robot, we have equipped a robot with own needs that autonomously trigger intentions and proactive behavior, and form the basis for understandable self-explanations. Previous research has shown that undesirable robot behavior is rated more positively after receiving an explanation. We thus aim to equip a social robot with the capability to automatically generate verbal explanations of its own behavior, by tracing its internal decision-making routes. The goal is to generate social robot behavior in a way that is generally interpretable, and therefore explainable on a socio-behavioral level increasing users' understanding of the robot's behavior. In this article, we present a social robot interaction architecture, designed to autonomously generate social behavior and self-explanations. We set out requirements for explainable behavior generation architectures and propose a socio-interactive framework for behavior explanations in social human-robot interactions that enables explaining and elaborating according to users' needs for explanation that emerge within an interaction. Consequently, we introduce an interactive explanation dialog flow concept that incorporates empirically validated explanation types. These concepts are realized within the interaction architecture of a social robot, and integrated with its dialog processing modules. We present the components of this interaction architecture and explain their integration to autonomously generate social behaviors as well as verbal self-explanations. Lastly, we report results from a qualitative evaluation of a working prototype in a laboratory setting, showing that (1) the robot is able to autonomously generate naturalistic social behavior, and (2) the robot is able to verbally self-explain its behavior to the user in line with users' requests.

## 1. Introduction

In recent years, the ability of intelligent systems to be understood by developers and users has been receiving increasing attention. Correspondingly, there is a rapidly growing body of work in the field of Explainable A.I. (XAI). Less work so far has been directed to this question in the field of robotics and human-robot interaction, although explainability is particularly important for social robots that are supposed to act autonomously in the vicinity of human users. Such robots are known to raise peculiar, often unrealistic attributions and expectations because users tend to anthropomorphize and ascribe intentionality to them (Wiese et al., 2017). This bears a risk as having difficulties to understand a robot's workings and, therefore, a lack of understanding its intentions has been described as a psychological hazard to the user (Salvini et al., 2021). Further, non-understandable robot behavior may be classified as a social error, leading to users perceiving the robot as less competent on a socio-affective level (Tian and Oviatt, 2021).

In order to prevent such negative effects of non-transparent robot behavior, it is of utmost importance to not only design for the most social robot behavior, but rather the most social *and* understandable behavior. That is, a robot's behavior should either be readily interpretable to the user, or it should be potentially explainable to the user, i.e., made interpretable through some additionally given explanations. We consider the case when the robot itself shall be able to produce a self-explanation of its own behavior, online and during a running human-robot interaction. Such a setting is different from classical XAI research, for what is to be explained (the agent's behavior) is hardly separable from the explainer (the robot) or delimitable from the interaction: the explanandum is being established within the interaction and evolves depending on which behavior is executed (and when) as well as how it is perceived by the explainee (the user). This, in turn, will generally depend on the user's prior experiences and expectations as well as general attitude toward social robots, all of which can change over the course of the interaction. Previous studies have shown that a robot's verbal explanations can increase the understandability and desirability of its behavior when rated by passive observers (Stange and Kopp, 2020). Here, we adopt the view that explaining is a social and interactive process (Rohlfing et al., 2021), and we argue that it is an essential next step to develop and investigate how social robots can generate and “co-construct” explanations with an active interaction partner, online and embedded in the evolving interaction context.

**Figure 1:**
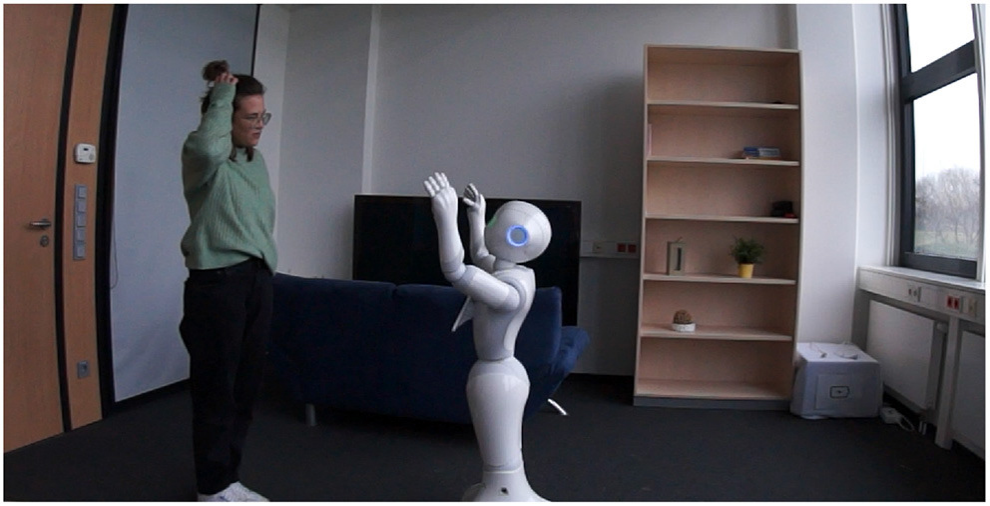
Interaction situation in which a puzzled user is looking for a behavior explanation by the robot.

In this article, we present the concept, implementation, and evaluation of an explainable social robot behavior architecture. The proposed architecture enables the social robot Pepper to autonomously interact with a user and to self-explain its own behavior, at the time and at the level of detail verbally requested by the user during the interaction. This work has been carried out within the VIVA project^1^, which aims at designing an autonomous, lively social robot for home environments (see Figure 1). Different from application scenarios in which human and robot collaborate in order to reach a shared task goal, behavior generation for social companion robots primarily aims to enable close social relationships. Correspondingly, social companion robot behavior generation, does not predominantly focus on the exact reproduction of the cognitive processes that lead to a human's behavioral task decisions and thus optimal collaboration, but rather on the generation of diverse and sociable behavior that fits the current interaction situation and may thus foster long term interest (Leite et al., 2013). In order to create a lively sociable robot presence, the VIVA robot is equipped with a motivational system that drives its behavioral decisions. The robot is able to autonomously decide on its behavior based on own *needs* that are connected to behavioral *strategies* (Stange et al., 2019). Some of these needs, e.g., for social contact or certainty, are influenced by events such as a user entering the room and lead to its rapprochement, while others are intrinsic, such as a need for energy linked to the robot's battery status. As a result, the robot can proactively select behaviors that may not be readily interpretable by the user and thus may need to be explained. Users shall be able to request an explanation from the robot at any time, when they don't understand the robot's behavior or simply when they want to know more about it. The architecture we present here supports this in two ways: (1) by organizing the robot's behavior generation process in terms of explainable attributes and categories, and (2) by adding a specific explanation generation model that introspectively retrieves information about the internal reasoning process and present it to the users in form of verbal explanations.

We start by discussing related work on robot architectures and explanations in human-robot interaction (Section 2). Based on this we will derive general requirements for explainable social robot architectures. In Section 3, we propose a user-centered framework for autonomous, explainable behavior generation and lay out a dialogic model for responding to explanation requests in human-robot interaction. In Section 4, we present an implemented interaction architecture, focusing on how the proposed autonomous behavior and explanation generation processes are realized. Subsequently, Section 5 reports insights from a first evaluation study, before discussing and concluding with a wrap-up as well as an outlook on future work (Section 6).

## 2. Related Work and Requirements

### 2.1. Interaction Architectures for Social Robot Behavior

Several architectures have been developed for social robots focusing on different aspects of social interaction (e.g., Breazeal et al., 2004; Laird et al., 2012; Baxter et al., 2013; Chao and Thomaz, 2013; Trafton et al., 2013; Adam et al., 2016; Moulin-Frier et al., 2018; Bono et al., 2020).

The work of Breazeal et al. (2004) explored a single-route architecture to generate attentive behaviors in a social robot. Visual cues such as eye gaze and pointing gesture were combined with verbal cues to detect which object in the environment the human interaction partner was referring to. Whenever the human shifted the gaze or referred to another object, the architecture triggered pre-defined behaviors to follow the gaze of the human or to point to the relevant object. Another single-route architecture is proposed in Tanevska et al. (2019) to dynamically adapt the level of engagement of a social robot based on social stimuli such as receiving a touch or seeing a face. A key component of the architecture is the module that computes a “comfort level” based on whether a touch or a face is detected. This serves as an internal drive that triggers pre-defined engagement or disengagement behaviors. The perception of social stimuli progressively increases the comfort level and the lack of this stimuli decreases the comfort level. When the comfort level exceeds the saturation limit or falls below the critical minimum, the robot disengages from the user and does not respond to social stimuli. This period is then used as a recovery phase to bring the comfort level back to the optimal range.

The above-mentioned single-route architectures defined associations between perceived information and the social behavior to be generated. In contrast, Lemaignan et al. (2017) proposed a deliberative architecture for human-robot interaction for shared task execution. This architecture identifies and integrates artificial cognitive skills such as theory of mind, visual-spatial perspective taking, geometric reasoning, knowledge representation and human-aware task planning, in order to enable the robot to model the beliefs and intentions of the human collaborator, assess the situation, and accordingly engage in joint actions (cf. Devin and Alami, 2016). Although several of these high-level capabilities are also required for social robots acting as companions, a striking feature of our robot is its animal-like liveliness, which is achieved through intrinsically motivated, needs-based behaviors, that are not always linked to the beliefs and goals of the human(s) inhabiting the shared physical space. Moreover, liveliness requires the integration of reactive, associative, and deliberative behaviors of different temporal resolutions and priorities, which necessitates multi-route architectures that are more specific than generic, theory-based cognitive architectures such as ACT-R (Anderson et al., 2004) and SOAR architecture (Laird, 2008, 2019), which primarily aim to model human cognition and are not geared toward the online processing of rich socially interactive behavior.

Multi-route architectures use multiple processing routes to generate appropriate behaviors. For example, in Adam et al. (2016), a dual-route architecture is proposed to generate emotional responses to verbal stimuli. It has a fast and reactive route that generates an initial emotional response, and a slow and deliberative route that reasons about the response by taking into account the verbal stimuli as well as the mental state of the robot. Generally, as the number of processing routes increases, the logic to integrate the different levels as well as the resulting behavior can get highly complex. Other works on social human-robot interaction did not propose an architecture but explored specific methods to realize key elements of an interaction, with the goal of increasing user engagement. In Chao and Thomaz (2013), Timed Petri Nets (TPN) were used to enable social robots to dynamically seize, yield, hold, and audit the conversational floor during 1-on-1 interactions with a human. In Park et al. (2019), multimodal affective cues were used as feedback to dynamically adapt the robot's storytelling strategy and thereby improve the engagement of the child.

As can be seen from the above examples, interaction architectures for generating social behaviors in robots involve several components and models that closely interact with each other (cf. Kopp and Hassan, 2022). Although the mentioned architectures were rather simple and focused on specific aspects of social behavior generation and interaction, it is evident that a combination of these solutions would be necessary for building a full-fledged social robot. Perception-based attentive behaviors, dynamic adaption to the preferences of the human interaction partner, possession of internal drives and emotions that dynamically influence behavior, naturalistic conversations involving dynamic turn-taking and accompanying nonverbal expressions, dynamic switching of behaviors depending on context, fluent integration of reactive, intuitive, and deliberative behaviors—all of these are essential for rich social human-robot interactions. However, this has the consequence that the internal logic for behavior generation will no longer be interpretable nor intuitive to the human interaction partner. Therefore, social robots need the capability to explain their behavior and, importantly, to provide reasons for it in a way understandable to naive human users.

In Han et al. (2021b), different ways to explain a robot's actions through nonverbal modalities were explored. More specifically, they tried to communicate the unreachability of an object in a hand-over task with the help of head shakes, head turns, and pointing gestures. The autonomous generation of these explanatory gestures was not discussed. Han et al. (2021a) leverage the hierarchical structure of Behavior Trees to automatically generate explanations for actions performed by the robot and to answer questions about how a complex task is performed. Although such algorithms are useful for task or action-specific explanations, they are insufficient to explain more general behaviors of an autonomous social robot. As mentioned earlier, autonomous behavior generally arises from an interplay of different components and models, and also through interactive processes that involve the user. To autonomously generate explanations of social robot behavior, we thus have to introduce necessary mechanisms (e.g., components and interfaces) at the conceptual level of the architecture. In this article, we propose and demonstrate the working of an interaction architecture that autonomously generates different types of verbal explanations at user's request.

### 2.2. Explanations in Human-Robot Interaction

Throughout this article we will use the term *interpretable* as defined in Ciatto et al. (2020), as “white boxes whose functioning is understandable to humans, also thanks to the expertise, resources, or tools.” If a system is not interpretable to a user (e.g., due to a lack of expertise or knowledge), we make use of *explainability*. As defined by Wallkötter et al. (2021), explainability of embodied social agents is their ability to provide information about their inner workings using social cues such that an observer (user) can infer how/why the embodied agent behaves the way it does. That is, an explainable model can be made interpretable through some additional explanations.

In the social sciences, explanations were described as social interactions early on: In his work on conversational processes and causal explanation, Hilton (1990) emphasizes the social nature of the explanation process, defining it as a three-place predicate: someone explains something to someone. Likewise, Rohlfing et al. (2021) summarize: An explainer explains an explanandum to an explainee. Research on explanations in human-robot interaction for a long time has looked at these different components and roles separately and not in an integrated fashion. In particular, previous work has focused on what a good explanation of robot behavior should entail, or how it should be presented with regard to how to best communicate specific action intentions or plans to increase a user's understanding.

Studies focusing on a robotic *explainer*, have for example suggested expressive motions (facial and bodily expressions) as a means of successfully communicating a robot's intentions to pedestrians (Mikawa et al., 2018), while Sado et al. (2020) conclude that for intuitive interaction one should aim at expressing explanations verbally, through natural language. Effects of the timing of a robotic explanations have been addressed by Zhu and Williams (2020), who investigated effects of explanations generated before actions were taken. Stange and Kopp (2021) showed that undesirable robot behavior should be explained after acting (as opposed to before). Han et al. (2021b) investigated a robot's arm movements and found that users prefer in situ behavior explanations. What is being explained, and thus the role of the *explanandum*, ranges from collaborative human-robot team work scenarios (Gong and Zhang, 2018) to explaining path plans (Chakraborti et al., 2020), to a robot's social behavior (Stange and Kopp, 2020). And lastly, whom it is being explained to (the *explainee*) has been shown to influence explanatory preferences on the basis of a person's characteristics such as age (Kaptein et al., 2017) or expertise (Ehsan et al., 2021b).

In recent years, a more socio-interactive view on the explanation process has emerged, focusing on the dynamic nature of explanations as emphasized for example by Morek et al. (2017), who describe explaining as a bidirectional process. Yet, a user-centered approach to explaining technical models and thus acknowledging the importance of adapting explanations to the recipient's (changing) needs throughout an interaction is by far not new. Fiedler (1999) used a cognitive architecture to plan dialogs for adaptive explanation generation based on a user model informing the generation architecture about previously gathered information on user preferences or capabilities. De Rosis et al. (1995) presented an implementation that not only accounts for eventual changes in the recipient's needs, but also takes the speaker's view into account. Ever since the relevance of including insights from the social sciences in XAI research has been emphasized (Miller, 2019), this socio-interactive view on explanations has experienced a resurrection. In Madumal et al. (2019), formalized an interactive explanation dialog model grounded in conversation data. Ehsan et al. (2021) stress the fact that explanations are socially situated and dynamically changing based on the goals and beliefs of both, explainer and explainee. They propose the concept of social transparency as a tool to incorporate the socio-organizational context into the explanation process of AI decisions. Rohlfing et al. (2021) describe explanations as a social practice, proposing a conceptual framework for studying the co-construction of explanations as a social and interactive process. And, recently, Matarese et al. (2021) highlighted the socio-interactive aspect in human-robot explanation situations and proposed a user-centered explanation framework that models the explanation process as an interaction between explainer and explainee.

### 2.3. Requirements for Explainable Autonomous Behavior Generation

Designing social robots that create long-term engagement with their human users requires a trade-off between consistency and variability of the robot's behaviors. The former is to ensure that users are familiar with the robot's behavior; the latter often results through adaptation/learning and is needed to sustain user interest. In both cases, it is crucial that users are able to interpret the robot's behavior in order to further and maintain users' trust toward the robot (Sheh, 2017). This can be achieved through enabling the robot to comprehensibly self-explain its behavior, in particular when it is unexpected or surprising to the user (Malle and Knobe, 1997).

The autonomous generation of lively, social behaviors in robots constitutes a complex technical challenge. Several different cognitive models and methods need to be integrated and run simultaneously and in close coordination with each other (Gratch et al., 2002). This requires an interaction architecture that integrates components for (1) sensing and processing multimodal information about the interaction partner and situational context, (2) modeling, monitoring, and managing internal drives and needs of the robot, (3) generating expressive and coherent needs-based multimodal behavior, (4) integrating behaviors of varying timescale according to context-based priorities, and (5) fluently adapting behavior in real-time to dynamic changes in the social interaction context. In addition to these already complex architectural and functional requirements, we demand such an architecture to be explainable. That is, we require it to support the autonomous generation of verbal explanations of the socio-interactive behavior it is producing. For this, behavior explanations and their generation must be co-designed with the interaction architecture. To that end an interaction architecture must fulfill the following requirements:

1. *Component-level inspectability*: Every component of the architecture that is causally involved in the robot's reasoning and action planning must be inspectable. This is necessary to enable the extraction and encoding of information relevant for explaining the role of these components in enabling social interaction.
2. *Interpretable inter-component communication interfaces*: The semantics of information exchange between different components should be well-defined in terms of their relevance to social interaction. These semantics should be reflected in the design of the inter-component communication interfaces in the architecture. This is necessary to enable the explanation of interaction between multiple components.
3. *Empirically validated explanation generation models*: The architecture must support empirically validated models for generating understandable and desirable explanations for socio-interactive behavior. This involves the definition of interfaces for delivering information relevant for behavior explanations. Crucially, the explanation generation models should be interpretable or explainable themselves, as the robot's explanation behavior can become an explanandum itself.
4. *Incremental behavior explanation generation*: Social interactions are highly dynamic, resulting in constant changes in information flowing through the architecture and in the actions performed by the robot. Therefore, in order to provide correct explanations without delay, it is important that the information necessary for explaining a behavior are generated and updated incrementally within the architecture, parallel to behavior generation.
5. *User-centered explanation delivery*: The architecture should be capable of delivering explanations according to the needs of the user. For this, it should be capable of understanding verbal requests from the user and dynamically adapting the explanation strategy. In addition, the architecture should support flexible dialog in order to ensure that the user requests can be served seamlessly, also within an ongoing conversation.

## 3. A Framework for Behavior Explanations in Social Human-Robot Interaction

In accordance with the above requirements, we propose a social robot architecture that enables the autonomous generation of rich, interactive behavior of the robot, while simultaneously supporting the flexible and autonomous generation of explanations for the produced behavior. In this section, we will first present our conceptual approach to how explanations of robot behavior can be given and interactively established in social human-robot interaction. The actual implementation of the architecture and the explanation generation model are then described in Section 4.

**Figure 2:**
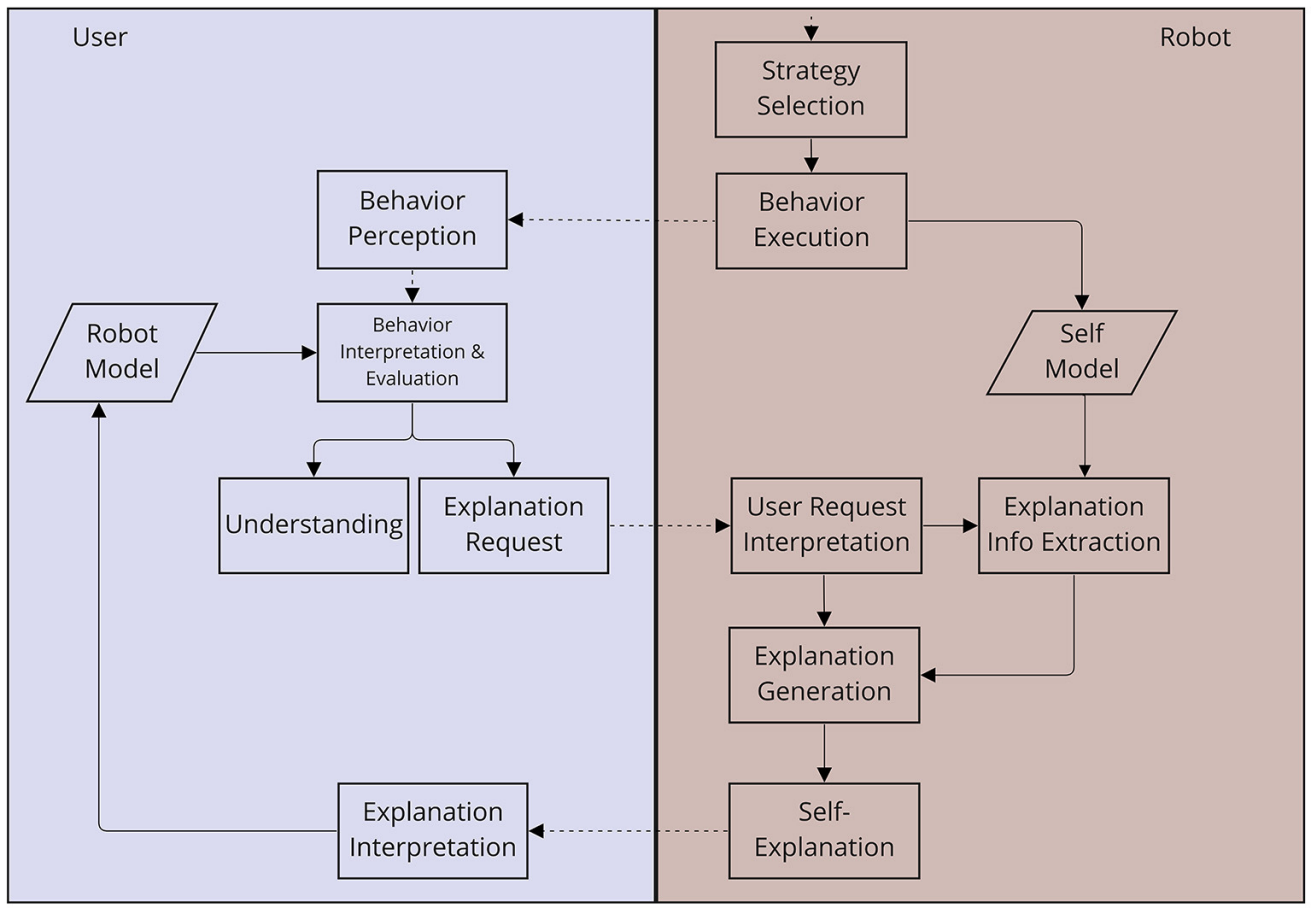
Overview of the proposed framework for robot self-explanations in social human-robot interaction.

Inspired by BDI (beliefs, desire, intention) principles and drive theory, our robot is equipped with internal needs that steer its behavioral choices (Stange et al., 2019). Based on its current needs and under consideration of the utility (impact on needs) and applicability (met preconditions) of possible behavioral strategies, the robot selects a strategy and executes the behavior(s) entailed. This straightforward behavior generation process is, at first, aimed at producing consistent, autonomous behavior which, due to its bio-inspired internal logic, should at the same time be potentially interpretable to human users. However, a behavior's degree of interpretability strongly depends on the situation it may occur in and the knowledge the user has about the robot's behavioral choices. More concretely, behavior that is interpretable for one user in one situation may not be interpretable in another situation or by another user. A user's need for explanation can thus emerge and change over the course of the interaction. We therefore propose an interaction-based, user-centered framework to lay down when and how a robot's social behavior should be explained (see Figure 2). It picks up on and extends Matarese et al.'s socio-interactive, user-centered view on providing explanations in human-robot interaction.

More specifically, we frame the interactive process of constructing an explanation as follows: A user continually *observes* a robot's behavior and tries to understand it (e.g., figuring out the agent's reasons). To that end, she *interprets* and *evaluates* the observed behavior based on her current *mental model* of the robot, which provides the basis for her expectations about the robot. The *observed behavior* may be interpretable resulting in the user's *understanding* and, possibly, acceptance of the behavior. In other cases, the observed behavior may not fit the user's current *robot model*, i.e., it is not fully interpretable and thus can result in an *explanation request*. Once such an *explanation request* is issued by the user, the robot *interprets* the *user's request* and *generates a behavior explanation* revealing information about the behavior or the reasons that led to it, as stored in a robot's *self model*. Upon receiving the robot's *self-explanation*, the user will update her mental *robot model* and *re-evaluate the behavior* based on the newly gained information, either resulting in a sufficient *understanding* of the behavior, or triggering further *explanation requests*. The robot must then be able to elaborate on its previous explanation, in a dialogic fashion that takes into account the user's current requests as well as the previous discourse. In this way, anchoring the robot's behavior explanations in the social interaction and enabling the user to evoke explanations *via* verbal inquiries allows them to actively co-determine when and what kind of explanation is sufficient. The underlying social interaction loop along with the different, involved communicative actions can most naturally and intuitively be implemented in a language-based dialog interaction.

**Figure 3:**
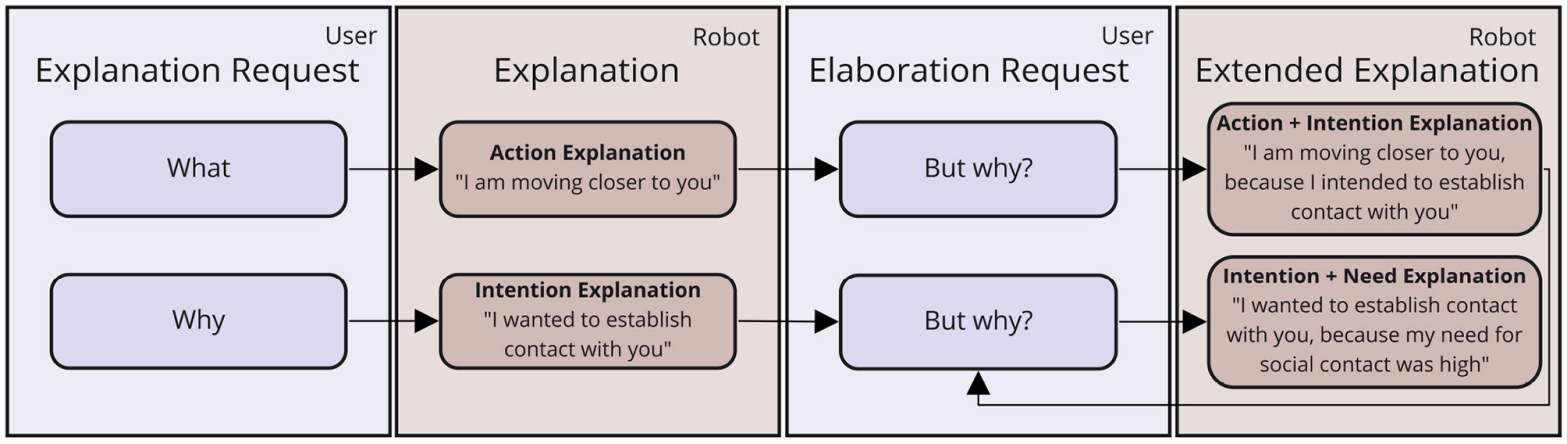
Flow model of explanation dialogs in socio-interactive human-robot interaction.

While the previously presented model provides a larger conceptual framework, we still need to define the specific forms of self-explanations the robot should produce in this model, and in response to which explanation requests of the user they should be given. Following the socio-interactive approach to explanations, we define a dialog flow model (see Figure 3) laying out *when* to provide *what* kind of explanation. That is, we differentiate between different kinds of user requests, explanation types and dialog contexts in which they can be employed. Following Madumal et al. (2019)'s grounded interaction protocol, we initially differentiate between what- and why-questions of the user. These requests mark the beginning of a hierarchically graded explanation dialog flow, in which different kinds of requests (for explanation or elaboration) are answered by the robot with different kinds of explanations. The latter are defined based on previous empirical studies in which different robot behavior explanations have been investigated in video-based online studies, with positive effects on the perceived understandability and desirability of robot behaviors (Stange and Kopp, 2020, 2021).

The different types of explanations included in the dialog flow are inspired by the different “domains” found by Malle in studies on how humans explain intentional behavior (Malle, 2004, 1999): (a) robot or user needs that the robot *desires* to fulfill, (b) *intentions* or strategies the robot selected to pursue, (c) concrete *actions* executed by the robot. In addition, to enable more complex and elaborate explanations, these domains of intentional behavior can be causally linked in structured explanations, leading to (d) intention formation-based explanations (linking need and intention), and (e) action selection-based explanations (linking intention and action).

Our dialog flow model lays out when and in response to which kinds of request these different kinds of explanations are to be given: In case the user asks the agent *what* it is currently doing, the robot gives a mere *action explanation*. If this answer does not lead to a sufficient increase in understanding and the user requests an elaboration, the robot will provide a combined *action selection-based explanation*. Similarly, if the user starts with a *why*-request, the robot will explain its *intention* and, in case of a subsequent elaboration request, follows up with a combined *intention formation-based explanation*. This explanation can also be given to elaborate upon an action selection-based explanation.

This dialog flow model of self-explanations can only be realized if the underlying robot architecture fulfills the requirements listed above. In particular, the different kinds of explanations need to be mapped to the structures and processes underlying the robot's decision-making and behavior planning. Here, the robot's self model (see Figure 2) plays a central role in generating these explanations: Episodic information about internal processes as well as external events must be encoded and stored in memory. For example, in our architecture, interaction episodes are defined on the basis of (i) user-initiated dialog sessions or (ii) robot-initiated plans of actions (Hassan and Kopp, 2020). This information is then fed as input to a module for dynamically generating behavior explanations that suit the user's need for explanation. How this is implemented in detail is described next.

## 4. An Explainable Social Robot Architecture

**Figure 4:**
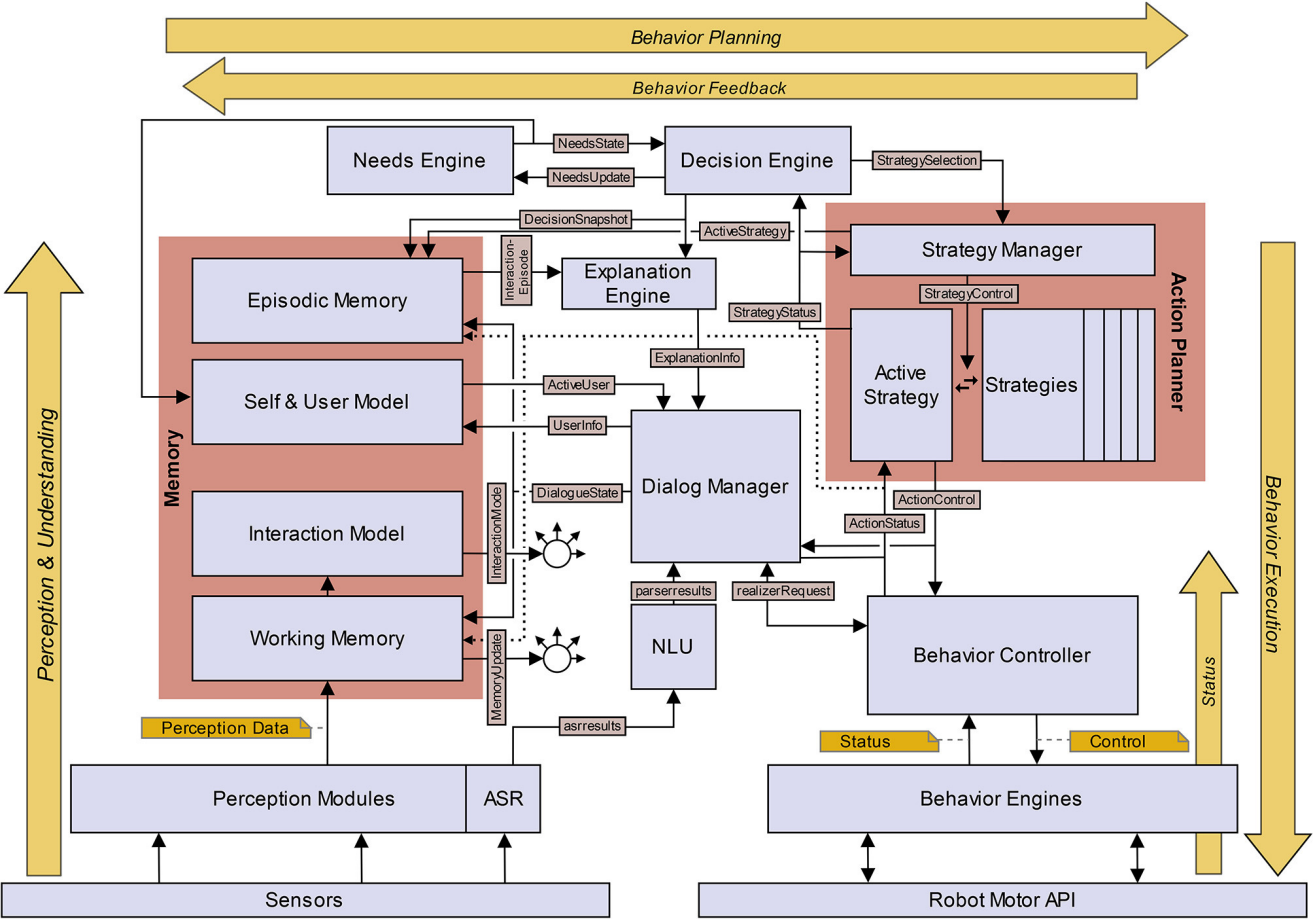
Schematic view of the explainable interaction architecture for an autonomous self-explaining social robot. The components are marked using rectangles and the most important communication interfaces between components are indicated by black arrows. Dotted arrows are used to enhance readability and have the same meaning as solid arrows.

To test the proposed framework for social robot self-explanations, we have implemented an explainable social robot architecture that fulfills the above-mentioned requirements. The architecture comprises different kinds of components: (i) modules for processing multimodal perceptual input for interpreting the active interaction context; (ii) modules involved directly in autonomous and needs-based socio-interactive behavior generation; (iii) modules involved in autonomous explanation generation; (iv) and modules needed for multimodal behavior realization. A graphical representation of the implemented interaction architecture is given in Figure 4. It is evident that several components of this architecture also appear partially in other social robot (control or interaction) architectures (see Section 2.1). The aim of this article is to show the co-design of socio-interactive behavior generation and explanation for a needs-based, lively social robot. Here, a first implementation of our interaction architecture (components and interfaces) is elaborated.

### 4.1. Perception and Interaction Context Modeling

#### 4.1.1. Perception

The *Perception* component receives data from the different sensors attached to the embodiment. These sensor data are processed to interpret relevant information about the state of the internal and external environment of the robot, and make this information available to other components. The perception component includes separate modules for handling different types of sensor data. For example, the *Interoception* module deals with the physical state of the robot, such as the state of its joints and batteries. The *Face Perception* module receives image data from the robot camera, and analyzes it to detect and recognize a face. It builds on the dlib^2^ library (King, 2009) and generates an embedding to represent the detected face with the help of a deep learning model based on ResNet^3^. This embedding is compared with embeddings from previous interactions, and an appropriate Universally Unique Identifier (UUID) is assigned. The estimation of the current position of the face and attributes such as age, gender, and emotion is performed using the SHORE^TM^ library (Küblbeck and Ernst, 2006; Ruf et al., 2011)^4^, which use classical machine learning models.

#### 4.1.2. ASR and NLU

Dialog processing begins with analyzing the speech input from the user to recognize the spoken words [*Automatic Speech Recognition (ASR)*]. Afterwards, the communicative intent is inferred from the recognized verbal phrases by the *Natural Language Understanding (NLU)* component. With the help of an incremental information processing strategy, the verbal phrases and intent are updated incrementally, as new speech input arrives. In the current implementation, we use Google ASR^5^ for automatic speech recognition and the open-source tool RasaNLU^6^, for intent classification. In line with the explanation dialogue framework proposed in Section 3), we have trained the NLU component to differentiate between three different explanation request intents: what-explanation requests, why-explanation requests and elaboration requests. To recognize named entities such as name, date of birth and hobbies of the user, we use Spacy^7^ and expression matching methods.

Note that, the machine learning models used for face detection, face embedding generation, speech recognition, and natural language understanding are black-box models, which could be made inspectable to some extent by applying suitable explainable AI methods (cf. Adadi and Berrada, 2018; Molnar, 2022; Requirement 1). However, causal explanation of the output of the above-mentioned machine learning models so far has not been relevant for the socio-interactive behavior explanations in our framework.

#### 4.1.3. Memory

The *Memory* component consists of modules for aggregating, fusing, or associating information from other components of the architecture, in order to infer higher-level information relevant for social interaction and social behavior generation. This component comprises separate modules for processing information at different temporal granularity and abstraction levels. The modules in the *Memory* component jointly describe and represent the active socio-interactive context. By virtue of the declarative and symbolic nature of these modules, they fulfill the inspectability requirement (Requirement 1).

The *Working Memory* processes only information and events from the present and the immediate past, to update internal memory variables having a short time span (e.g., face_in_view, gaze_running, user_greeted, etc.). The preliminary implementation uses a queue to store heterogeneous information for a short period of time and retrieve the last known information not older than a specific time interval. The *Episodic Memory* integrates events and actions into an interaction episode. It tracks and updates the ongoing interaction episode and persistently stores past interaction episodes (see Hassan and Kopp, 2020) for details). The ongoing interaction episode provides information about the active robot behaviors and actions, which can then be used by the robot for generating the action explanation. In the initial implementation, each interaction episode is represented as a collection of key-value pairs and all past interaction episodes are stored in a JSON file.

**Figure 5:**
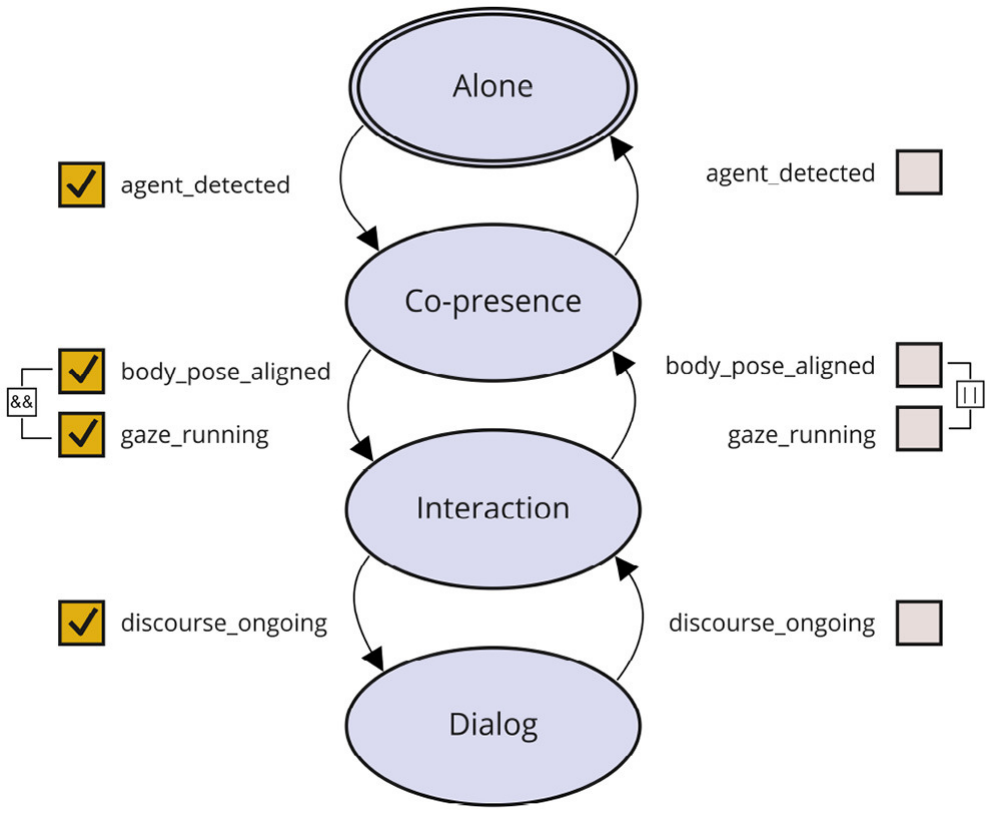
State machine describing possible interaction modes along with their transitions.

The *Interaction Model* implements a state machine which is used to classify and keep track of the current human-robot interaction situation (see Figure 5). This state machine comprises four interactions modes, namely, *Alone, Co-presence, Interaction*, and *Dialog*. The transitions between these states are triggered by Boolean-valued internal memory variables. The interaction modes can be used as preconditions to systematically enable behavioral strategies suited to the current socio-interactive situation. The *User Model* builds profiles of users based on information gathered *via* face perception and natural language understanding. In the current implementation the *User Model* stores UserInfo (UUID) and data extracted from the user's face *via* the *Face Perception* module, such as age and gender, emotional state, and gaze direction. It further provides the infrastructure to record the user's name, birthday, hobbies and interests, gathered and updated *via* verbal interaction with the user. The stored information about the *ActiveUser* are at disposal for other modules and, e.g., used by the *Dialog Manager* to greet a specific person with their name. The user profiles are stored in persistent storage (currently, as a JSON file) and retrieved based on the identity of the person interacting with the robot. The *Self Model* manages the robot's internal states, for example, its emotional state, which is represented within a Valence-Arousal-Dominance (VAD) space and is influenced by own needs as well as the user's emotions.

### 4.2. Autonomous Needs-Based Socio-Interactive Behavior Generation

#### 4.2.1. Needs Engine

The *Needs Engine* models the state of needs of the robot and of the user. Currently, there are five robot needs (*certainty, energy, social contact, relaxation*, and *entertainment*) and two user needs (*contact* and *rest*). The needs are modeled as continuous values between 0 and 1, and grow linearly over time according to their respective growth rate. These needs can also be influenced by changes in the internal memory variables (e.g., agent_detected increases the robot's *social contact* need). The *Needs Engine* periodically publishes the current values of the robot's and user's needs within the architecture. Based on these values, the *Decision Engine* dynamically selects an appropriate high-level, socio-interactive behavior (or, *Strategy*) that would minimize the overall needs. The needs are updated accordingly by the *Decision Engine*, when the selected strategy completes execution. In the current implementation of the *Needs Engine*, each need is modeled independently of the other needs. This makes it easier to extend or update the list of modeled needs in the future.

#### 4.2.2. Decision Engine

The *Decision Engine* is in charge of selecting the next *Strategy* (or, high-level behavior) to be executed based on the current state of needs (provided by the *Needs Engine*). In each decision-making cycle, only those strategies that are relevant for the current interaction mode (given by the *Interaction Model*) are considered as candidates. Each *Strategy* *S* has an estimated average execution time *t*_*S*_ and an expected impact **I**_*S*_ on the needs of the robot and the user. In each cycle, the *Decision Engine* constructs all possible plans that can be executed within a specific time interval in the current state (specified by the variables in the *Working Memory*). A plan **P** is an ordered sequence of one or more of the candidate strategies. An objective function *f*(**P**) measures the effectiveness of a plan based on the impact that each strategy *S* in the plan is expected to have on the needs of the robot and the user. Using the needs impact vector **i**_*S*_ and the average execution time *t*_*S*_ of *Strategy* *S* as well as the vector of growth rates of needs **g**_**N**_, the expected change (growth or decay) Δ**n**_*S*_ in the state of needs after the execution of *Strategy* *S* is calculated (see Equation 1)^8^. The expected state of needs after the execution of the plan (**n**_**P**_) is calculated by aggregating the changes contributed by each strategy in the plan and adding them to the state of needs at the start of the current decision-making cycle **n**^(0)^, as shown in Equations (2) and (3). The objective function then computes a weighted sum of squares of these expected needs (see Equation 4). The weights vector **w**_**N**_ encodes the relative importance of individual needs and thus assist in dealing with conflicting needs^9^. Currently, the weights are identical for all users, but could be adapted in the future to match user preferences and traits, which could be learned and recorded by the *User Model*.


(1) $$\begin{array}{l} {\Delta \text{n}}_{S}={\text{i}}_{S}+t_{S}{\text{g}}_{n} \end{array}$$

(2) $$\begin{array}{ll} {\Delta \text{n}}_{\text{P}} & =\underset{S\in \text{P}}{\sum }{\Delta \text{n}}_{S} \end{array}$$

(3) $$\begin{array}{ll} {\text{n}}_{\text{P}} & ={\text{n}}^{\left(0\right)}+{\Delta \text{n}}_{\text{P}} \end{array}$$

(4) $$\begin{array}{l} f\left(\text{P}\right)={{\text{n}}_{P}}^{T}({\text{n}}_{P}◦{\text{w}}_{N}) \end{array}$$

A small value for *f* indicates that the plan can effectively decrease the overall and the most influential needs. The plan having the smallest value for f is selected as the best plan, and the first *Strategy* in this plan is the next strategy that will be executed. This decision is recorded in the form of a “decision snapshot” that specifies the selected strategy along with the robot and user needs at the beginning of the decision-making cycle. This causal relationship between needs and active high-level behavior can be subsequently used for generating intention explanations and extended explanations based on intention and needs (see Section 4.3). The selected *Strategy* is sent to the *Strategy Manager*, which then starts this strategy after terminating any currently active strategy. Thus, in the current implementation, the *Strategy Manager* ensures that only one strategy is active at a time.

By basing the decision on plans, it is expected that the chosen strategy may make other advantageous strategies feasible in the future. In order to avoid behavior repetition, the previously selected strategies are excluded from successive decision-making cycles for 30 s. A new decision-making cycle (or, re-planning) is started when no strategy is active or when the state of needs increased significantly after the last strategy selection. In this way, the robot autonomously chooses its behavior under diverse and changing external events and internal needs.

#### 4.2.3. Action Planner

A set of predefined *Strategies* in the *Action Planner* component determine the available high-level socio-interactive behaviors. A *Strategy* is associated with an intention, e.g., initiating contact with a user (*StrategyInitiateContact*), and describes a high-level behavior or action plan to realize this intention. It may consist of ordered subgoals (post-conditions) that are fulfilled by elementary actions. The elementary actions can be either high-level dialog behaviors (e.g., greet_user, acquaint_with_user) that are forwarded to the *Dialog Manager*, or non-dialog behaviors (e.g., approach_user) which are sent directly to the *Behavior Controller* for realization. The execution of these actions may require certain preconditions to be fulfilled. Pre- and post-conditions are Boolean-valued internal memory variables that reside in *Working Memory*.

Currently, *Strategies* are implemented in the form of Behavior Trees (Colledanchise and Ögren, 2018) using the PyTrees library^10^. Behavior Trees are hierarchical arrangements of nodes representing conditions, actions, or control logic for executing child nodes (e.g., sequential, parallel, selective execution). Of particular relevance to the present work are the selector and sequence nodes. A selector node executes a child node on its right, only if all the child nodes on its left have failed. If at least one of its child nodes is successful, then the selector node is also successful. A sequence node executes a child node on its right, only if all child nodes on its left have succeeded. It is successful only if all its child nodes are successful. The explicit structure of the Behavior Trees makes the high-level behaviors inspectable and, consequently, explainable (Requirement 1).

**Figure 6:**
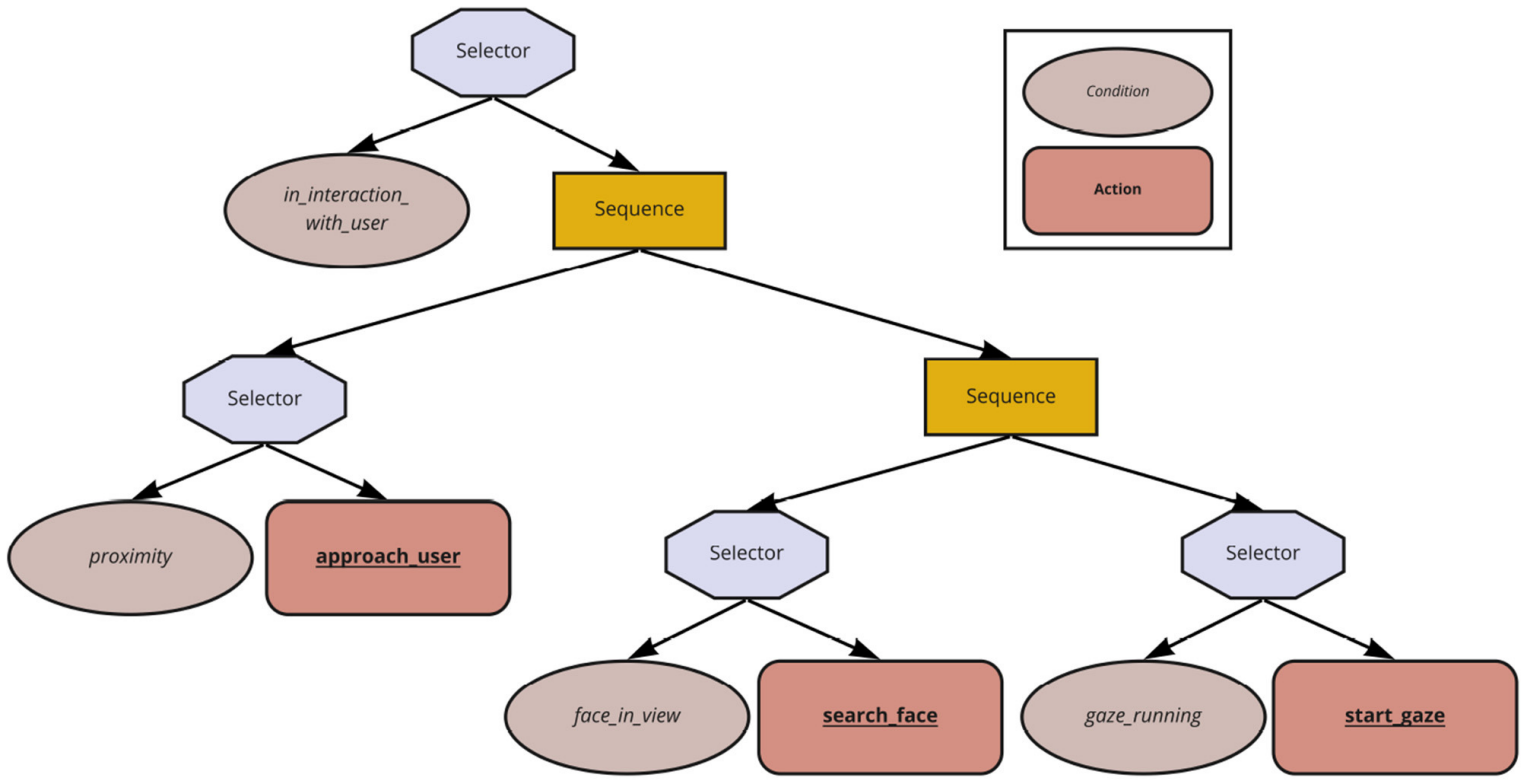
Example Behavior Tree for *StrategyInitiateContact*: The octagons represent selector nodes, rectangles represent sequence nodes, ellipses represent condition nodes, and rounded rectangles represent action nodes.

In Figure 6, the Behavior Tree representation of *StrategyInitiateContact* is shown. A selector node initiates the robot to drive toward the user (action: approach_user) only if it is not already in the proximity of the user (condition: proximity). The sequence node above this selector node ensures that, once the robot is in the proximity of the user, the right subtree may be executed, leading the robot to have eye contact with the user and thus initiating the interaction. Usually, each subtree follows the concept of either a condition being true or executing the action/subtree that would eventually make the condition become true.

Each *Strategy* (high-level behavior) is associated with a set of preconditions that should hold before a behavior can be initiated, and a set of post–conditions that are fulfilled when the behavior has been successfully executed. Pre-conditions of strategies can be either an interaction mode that is determined by the *Interaction Model* or an internal memory variable that is published by the *Working Memory*. Each strategy is also annotated with a vector denoting its impact on the needs of the robot and the user, when executed. Every time a strategy is successfully completed, its average duration attribute is updated. There is also a time-out duration associated with the strategy to recover from potential execution-time issues.

All available *Strategies* and their key attributes are listed in Table 1. During start-up of the architecture, all *Strategies* are initialized and other components (e.g., *Decision Engine, Explanation Engine*) are informed about the available strategies and their attributes, which includes the strategies' needs impact vectors. This information is used to generate the intention + need explanation, verbalizing the need the selected strategy has the highest impact on. In the future, new strategies can be added without having to change existing strategies.

**Table 1:** Overview of all the *Strategies* defined in the current implementation of the proposed interaction architecture. The table shows the preconditions and post-conditions of the strategies, their comprised actions, and the robot and user needs that are influenced by their execution (! denotes the negation of the mentioned variable).

**Strategy name**		**Strategy idle**	**Strategy initiate contact**	**Strategy greeting**	**Strategy acquaint with user**	**Strategy loosen up**	**Strategy charge battery**
Preconditions	Memory variables			! User_greeted	User_greeted ! Acquainted_with_user	! Loosened_up	Energy_critical
	Interaction mode	Alone Co-presence	Co-presence	Interaction	Interaction	Co-presence Interaction Dialog	
Postconditions	Memory variables		Gaze_running Face_in_view Proximity Body_pose_aligned	User_greeted	Acquainted_with_user	Loosened_up	Battery_charged
Actions		Idle_gestures	Approach_user Search_face Start_gaze	Greet_user_via_dialog	Acquaint_with_user_dialog	Stretch	Move_to_charging_station Use_charging_station
Needs impact	Self		Social contact	Social contact Certainty	Social contact Certainty	Relaxation	Energy Relaxation
	User	Rest	Contact	Contact	Contact		

#### 4.2.4. Dialog Manager

Any verbal communication with the user is managed by the *Dialog Manager*. It handles both communicative actions triggered by the active strategy as well as user-initiated conversations and explanation requests. We employ the previously developed flexDiam system (Yaghoubzadeh and Kopp, 2017) to plan the next phrases to be spoken by the robot based on the active dialog context and the user's communicative intent. In flexDiam, dialog is considered to be organized hierarchically according to *Issues* that are addressed by the user and the robot cooperatively. Several *Issues* can be active simultaneously in the background and are triggered upon request, enabling an interactive dialog and yielding the possibility to react to off-topic user requests. For example, the user is always able to ask the agent to repeat its last utterance, or to end the dialog and leave the conversation. Upon completion of an issue, higher-level issues that have not yet been completed are resumed. The agent is thus able to handle interruptions or interjections. Issues that wait for a response are re-triggered if no answer has been received within floor time allotted to the user.

In the current implementation, the following issues are realized: A *GreetingIssue* and an *AcquaintIssue*, which includes child issues that gather information about the user (*GatherName, GatherBirthday*, and *GatherHobbies*) or alter information already stored about the user (*CorrectingIssue*); an *AboutUserIssue* that is used to answer questions about the information the robot has stored about the user; an *AboutVivaIssue* that is used to share information about the robot itself; a *HowAreYouIssue* that is used to answer questions about the robot's emotional state; an *EmotionIssue* that is used to inform about the user emotion the robot has recognized; and finally, an *ExplanationIssue* that is triggered when recognizing a user explanation request. The *ExplanationIssue* evokes the generation of a robot self-explanation according to our dialog flow model (details described in Section 4.3).

FlexDiam captures the sequential structure of the dialog on a timeboard, on different tiers related to different elements like words, phrases, floor, or messages to and from other components of the architecture. Information about the current dialog state, e.g., whether a discourse is ongoing (an *Issue* is open) or not, is communicated to the *Working Memory*. Likewise, user information obtained through dialog is sent to the *User Model*. On the other hand, flexDiam receives data from other components, e.g., to respond to user queries or to exploit knowledge stored in the *User Model*. All *Issues* employ an embedded *Natural Language Generation (NLG)* component to generate utterances and determine accompanying nonverbal behaviors, which are then sent to the *Behavior Controller* for realization.

### 4.3. Autonomous Explanation Generation

#### 4.3.1. Explanation Engine

**Figure 7:**
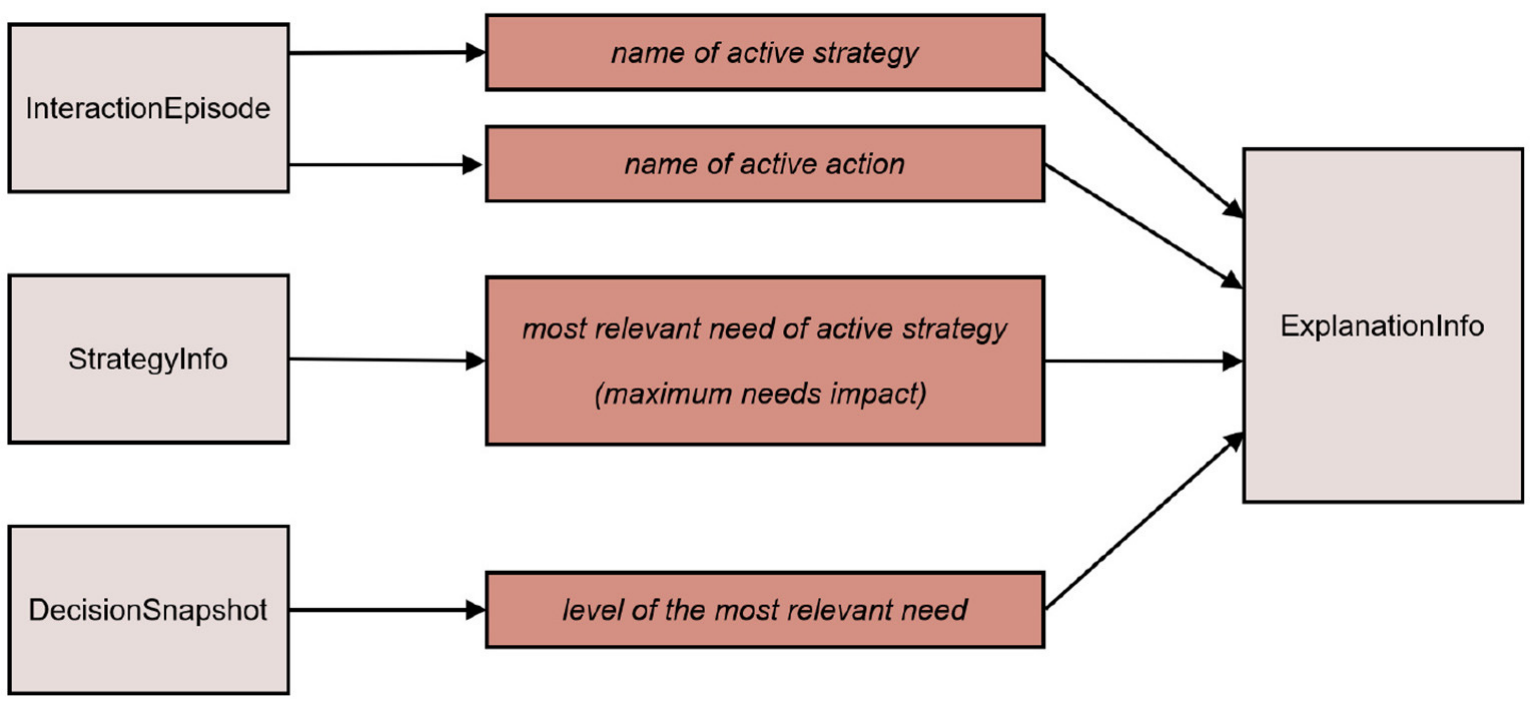
The content of ExplanationInfo (middle column) and the source messages from which the relevant information are extracted (right column).

**Figure 8:**
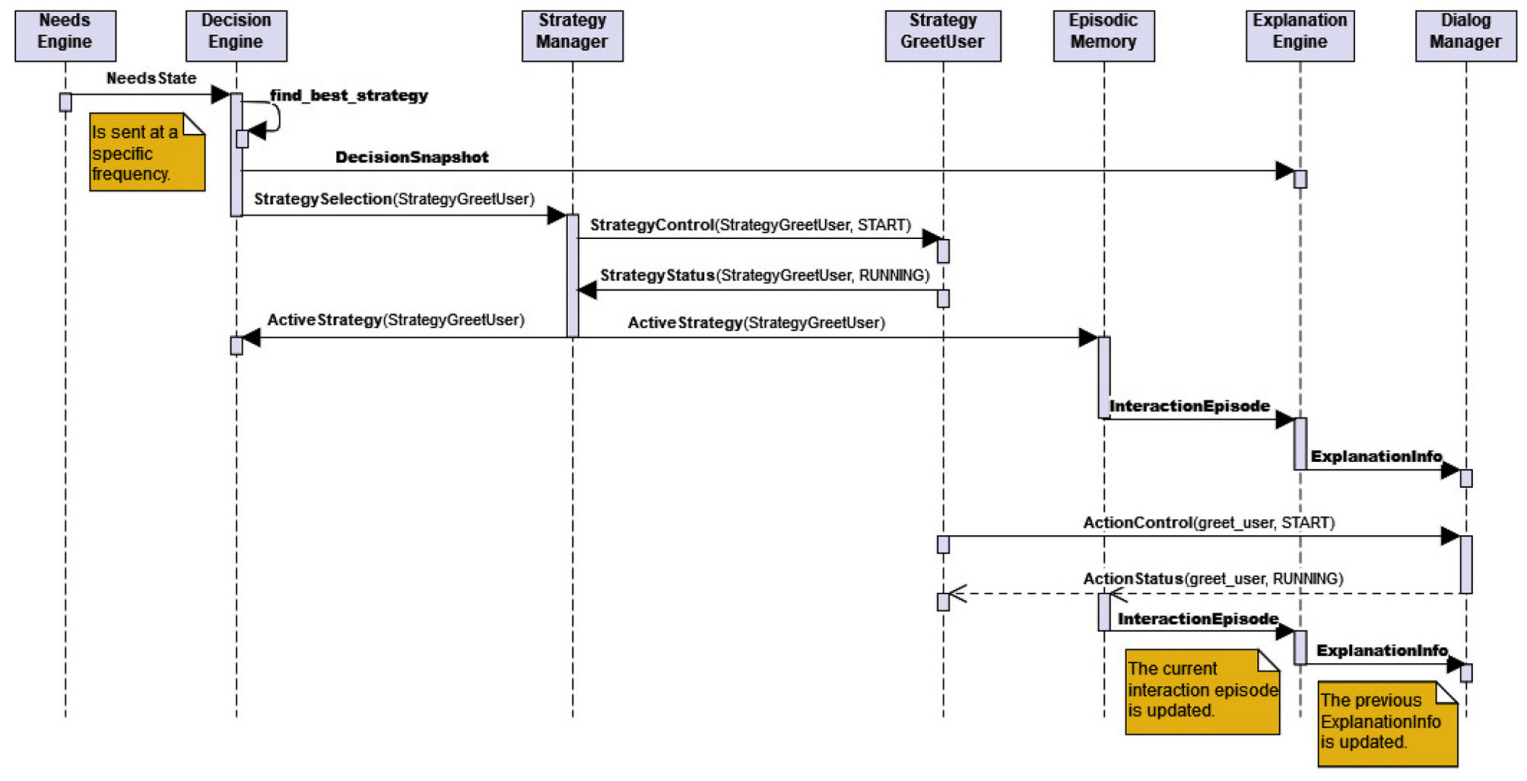
A sequence diagram showing the interaction between different components of the architecture to initiate a *Strategy* (high-level socio-interactive behavior) and simultaneously extract the information necessary for explaining this behavior (Requirement 4).

The *Explanation Engine* collects all information that are potentially necessary for explaining the current behavior of the robot, as proposed in the explanation dialog flow model (Section 3). This information is gathered from different components in the architecture. The *Decision Engine* sends a “decision snapshot” to the *Explanation Engine*, containing the selected strategy along with the specific robot and user needs at the time the strategy was selected for execution. The needs impact vector associated with each *Strategy* is collected when the strategies are initialized at start-up (*via StrategyInfo* message). The currently active dialog-based or non-dialog-based action (or, behavior) is specified in the current *Interaction Episode* which is issued by *Episodic Memory*. The *Explanation Engine* puts these pieces of information together into the structure *ExplanationInfo* and makes it available to the *Dialog Manager* so that it can explain the current behavior, when the user requests for it (as described below). Figure 7 shows how an instance of ExplanationInfo is initialized or updated. Any changes to any information relevant for ExplanationInfo are updated incrementally and notified by the respective components immediately (Requirement 4). This is illustrated in the sequence diagram in Figure 8, where the incremental updates are triggered by a newly started action. It also shows that the behavior generation and the extraction of information relevant for explanation take place hand-in-hand.

#### 4.3.2. Explanation Generation

The *ExplanationIssue* is activated in flexDiam whenever an explanation request is identified by the *NLU*. The explanations are then constructed according to the empirically validated explanation types described in Section 3 (Requirement 3). The type of explanation requested by the user (i.e., what or why explanation) is stored in the context and used by the NLG component to generate an explanation phrase based on the explanation type and the last received explanation information from *ExplanationEngine*. If an explanation request is followed by an elaboration request, the *ExplanationIssue* fetches the last handled explanation type from the context and commissions an appropriate elaboration based on this information. The corresponding explanation phrases are generated by the NLG module using templates for the different explanation types. Based on the provided information, the NLG inserts sub-phrases into these templates. An action-based explanation triggered by what-questions uses a template like “I am [action_name].” For example, for the action greet_user, [action_name] is substituted with the sub-phrase “greeting you” and for drive_to_charging_station, it is substituted with “driving to the charging station.” An intention explanation inserts the name of the active strategy into the template “I wanted to [strategy_name].” For example, for the strategy *StrategyInitiateContact*, the sub-phrase is “establish contact with you.” The extended explanation inserts multiple phrases into a template. Precisely speaking, it repeats the last delivered explanation and extends it with causal information about the intention or the most relevant need. Extended explanations include “I am [action_phrase], because I wanted to [strategy_phrase].” and “I wanted to [strategy_phrase], because my need for [relevant_need_name] was [relevant_need_level].” The sub-phrases for substituting [relevant_need_name] are identical to the name of the need. The continuous-valued needs are discretized into three levels by applying two thresholds (0.3 and 0.7). Accordingly, the level of the need is termed as “high,” “medium,” or “existent.” All verbal explanation phrases are accompanied by a hand gesture of the robot.

In sum, the explanation dialog flow model proposed in Section 3 is implemented by the issue structure of the *Dialog Manager* and, in this way, enables explaining and elaborating according to users' needs for explanation evolving in the interaction. The interplay of the input and output processing modules, the socio-interactive behavior generation modules, and the explanation generation modules, enables users to request and receive incrementally updated self-explanations of the robot, at their preferred time or level of detail during an ongoing dialog (Requirements 4 & 5).

### 4.4. Multimodal Behavior Realization

#### 4.4.1. Behavior Controller

This component is responsible for coordinating and managing the execution of communicative and non-communicative robot behaviors, which are either the elementary actions of the active *Strategy* or utterances requested by the *Dialog Manager*. The *Behavior Controller* instantiates any requested behavior and keeps track of its execution status. In addition, it coordinates the allocation of hardware resources between individual behavior instances and acts as a proxy for communication between individual behaviors and other components of the architecture. Multimodal communicative behaviors requested by the Dialog Manager, are dialog acts specified in the Behavior Markup Language (BML) (Kopp et al., 2006) syntax. These BML descriptions may contain the text to be uttered along with accompanying non-verbal behaviors. In our use case, every utterance is marked with a desired communicative intent (speech act), namely question, explanation, affirmation, denial, or greeting. When an utterance is received, a behavior is instantiated that selects a gesture corresponding to the communicative intent and then triggers *Speech* and *Gesture Engines via* control commands. Non-communicative behaviors (e.g., approach_user) that may be requested by the active *Strategy* or by the user directly, are realized using Locomotion and Gesture Engines.

#### 4.4.2. Behavior Engines

The transformation of individual behavior elements into embodiment-specific commands is done *via* dedicated behavior engines. The *Speech Engine* handles the commands for verbal output, the *Gesture Engine* handles body animations and individual joint movements, and the *Locomotion Engine* handles the rotation and translation of the whole robot in space. The engines provide the execution status of commands in individual status messages, which are then distributed to higher-level components via the *Behavior Controller*. The implementation of these engines differs depending on the used robot. In our use case, to communicate with the robot Pepper^11^, the engines use the NAOqi Bridge, which interfaces between ROS and Aldebaran's NAOqi^12^. In this way, the *Speech Engine* can use Pepper's TTS, the *Gesture Engine* can execute pre-defined animations and joint motion trajectories safely, and the *Locomotion Engine* can drive the robot at safe velocities. We also use the Basic Awareness ability of NAOqi to realize the gaze behavior and enable the robot to react to people and movements in the environment (sound and touch stimuli are currently ignored). Although the *Behavior Engines* and the *Behavior Controller* are inspectable due to their declarative nature (Requirement 1), the models used for realizing the behaviors on the hardware might not always be available for inspection, due to the proprietary nature of those libraries.

### 4.5. Communication Interfaces

In the current implementation of the architecture, communication between sensors, actuators and other hardware components takes place *via* the ROS middleware. The communication between all other components, modules, and models uses the Python 3 implementation of an incremental communication framework (Schlangen et al., 2010) (IPAACA^13^) with MQTT as the transport protocol. As shown in Figure 4, the names of the interfaces convey the semantics of the information exchanged between components (Requirement 2). Moreover, the information transported in the IPAACA message payload is represented in the JSON format, making it easy to interpret the content of the messages and to trace the causal structure of the behavior generation process within the architecture (Requirement 2). Except some parts of the perception component and low-level hardware drivers, all parts of the architecture have been implemented in Python 3. Furthermore, modularity, extensibility, and portability were key considerations during the implementation of the architecture. Except the low-level components that interface or communicate with sensors and actuators (i.e., *Perception modules* and *Behavior Engines*), all other components are independent of the physical embodiment and can be easily ported to other social robot or virtual agent platforms.

## 5. Evaluation

To test the proposed framework for social robots' behavior explanations, as well as its implementation in the presented architecture, we have carried out a first evaluation in live human-robot interactions in a lab setting. More specifically, a qualitative evaluation was designed to investigate if the first working prototype fulfills both, the

1. architectural and functional requirements, *via* evaluation of the robot's ability to (1) sense and process multimodal information about the social context, (2) generate naturalistic and coherent multimodal behavior, and (3) fluently adapt its behavior to dynamic changes in the social interaction context
2. explainability requirements, *via* evaluation of the robot's ability to deliver explanations in parallel with generating behavior (Requirement 4) and according to the needs of the user (Requirements 3 & 5), indirectly validating adherence to Requirements 1 & 2.

### 5.1. Materials and Methods

The evaluation included an acquaintance interaction between a human user and the social robot “Viva” (here, Pepper) situated in a laboratory living room setup. Throughout the interaction, the robot was entirely controlled by the above-described behavior generation architecture. Ethics approval was obtained from the ethics committee at Bielefeld University and COVID-19-related hygiene and safety precautions were taken.

**Figure 9:**
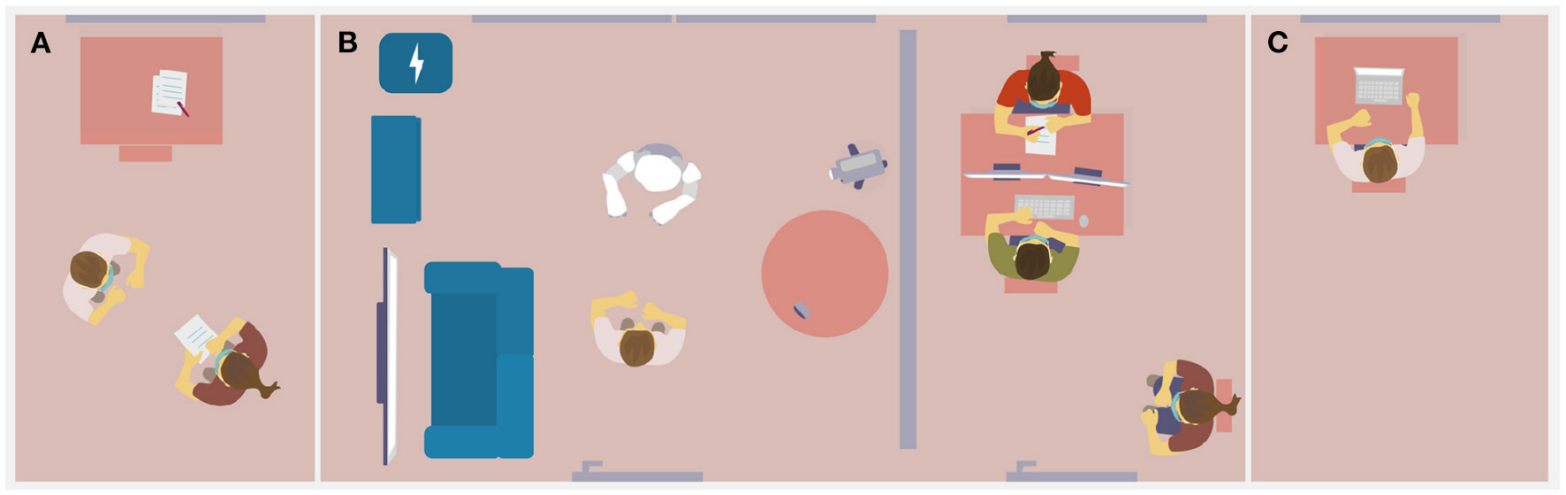
The experimental setup and procedure including **(A)** the participant being introduced to the robot and the task by the experimenter, **(B)** the actual human-robot interaction supervised by technicians and, **(C)** a post-interaction survey taken in a separate room.

The procedure of the study is depicted in Figure 9. Upon arrival, participants were invited to read the participant information and consent to the data policy. Thereafter, participants received verbal instructions about the robot being developed to live with people in their homes, being able to move in the room and to interact with users vis-à-vis. They were then informed that the research objective was to investigate how Viva's behavior is perceived. Participants were told that they had a few minutes to get to know Viva and should try to find out how the robot generated its behavior over the course of the interaction. They were explicitly told that they should find out what the robot was doing and which reasons it had for its behavior, and that they could ask the robot about this. Finally, participants were informed that the investigator would knock at the door after about 5 min, which was their signal to say goodbye and leave the room and that they would be asked to complete a survey to describe their impressions and insights with regard to Viva's behavior afterwards. Upon entering the room they should move to a position marked on the floor. The last instruction was that participants should try to speak loudly and clearly and in simple, short sentences in order for the robot to understand them well and that they could see when Viva was listening, by a green light in its eyes. The experimenter then accompanied the participant to the lab living room, started the camera, instructed the participant to take off the mask in order for the robot to see their face, and left the room. As depicted in Figure 9B, the lab living room was furnished with a sofa and TV screen, a cupboard, the robot's charging station, as well as a table and SoftBank's Pepper robot. A microphone, used for users' speech input was placed on top of the table and the interaction was filmed by a camera placed behind the table.

The interaction was not scripted but unfolded through the robot interacting autonomously with the participants. That is, the robot was able to select strategies, execute the corresponding behaviors, and communicate with the users *via* dialog. The following strategies were available to the robot: *StrategyIdle, StrategyInitiateContact, StrategyGreeting, StrategyAcquaintWithUser, StrategyLoosenUp, StrategyChargeBattery*. As the robot's perception did not yet account for recognition of a user's presence in the room, this memory value was manually set to true, once the experimenter had left the lab and the participant was alone in the room with the robot, setting the preconditon for the *StrategyInitiateContact*. Due to the restricted interaction time during which the robot was fully operable, the robot's energy status was not updated based on its actual battery status. Therefore, in order to evoke the selection of this strategy, experimenters decided to manually send an event that increased the robot's need for energy after some time. An example video of the interaction (with the researcher in the participant role) can be accessed online^14^.

A protocol of the interaction including all selected strategies and dialog events was stored. In the post-interaction questionnaire (implemented in soscisurvey^15^), participants' input on how they perceived the robot's behavior and explanations was gathered *via* quantitative items, as well as open questions. Robot's behavior was rated by participants with regard to intentionality, understandability, surprisingness, and desirability. Regarding the robot, ratings of likeability (five items, adapted from Reysen, 2005), multidimensional trust (five items, adapted from Bernotat et al., 2017), and intelligence (five items from Bartneck et al., 2009) were gathered. The robot's behavior explanations were rated with regard to their general understandability, the extent to which they adequately justify the behavior, as well as help the user understand why the robot behaved as it did. All items are gathered on 7-point Likert scales. Further, participants were invited to report moments they found particularly natural, unnatural, or striking and give feedback on how to improve the interaction and the robot's functionality in the future.

### 5.2. Analysis and Discussion of Results

In total, 12 participants took part in the interaction with the robot. One interaction had to be canceled due to a system failure and was thus excluded from the analysis. This led to a total of *N* = 11 complete interactions and datasets. Participants (six males, five females) were between 23 and 34 years old (*M* = 27.64, *SD* = 3.5). 5 participants were students and the majority of the participants had not interacted with a social robot prior to this interaction (*N* = 8). Quantitative data was analyzed using JASP^16^. The average duration of interaction was 07:09 min (min 05:20, max 09:26).

#### 5.2.1. Autonomous Social Behavior Generation

In order to analyze fulfillment of the architectural and functional requirements (1) *sensing and processing multimodal information about the social context* and (3) *fluently adapt its behavior to dynamic changes in the social interaction context*, the data stored in the interaction protocol was analyzed: The robot selected adequate behavioral strategies based on the current interaction mode. To start off the interaction manually, the *StrategyIdle* was set to success and the memory event (agent_detected) was sent to the robot once a participant was present and after the experimenter had left the room. The robot was able to process this information, leading to a change in its interaction mode from *Alone* to *Co-Presence* and triggering the robot to approach the user. In all interactions, the robot was idling (*StrategyIdle*) while *Alone* and once detecting the user in the room, switched to interaction mode *Co-presence* and reliably decided for *StrategyInitiateContact* and *StrategyGreeting*. Subsequently, if the robot was able to detect the user's face, it switched to the interaction mode *Interaction* and selected the now available *StrategyAcquaintWithUser*. If not, the robot remained in the interaction mode *Co-Presence* and started with *StrategyLoosenUp*, followed by re-starting the gaze behavior and selecting the *StrategyAcquaintWithUser*, if the users' face was in view now and the interaction mode thus changed to *Interaction*. Upon reception of the energy_drop event, the robot's need for energy increased, leading to the selection of the *StrategyChargeBattery*. The robot was further able to sense and process verbal input, as well as visual input about the user's face, enabling it to successfully gather user information in the acquaintance dialog during every interaction.

An assessment of whether the implemented architecture enabled (2) *naturalistic and coherent multimodal behavior generation* is best given from the users' feedback: Due to the small amount of participants and a high variability between the interactions that unfolded autonomously, results from the analysis of the questionnaire data should be regarded with care, indicating at best a general direction. Mean ratings of the behavior and robot ratings were analyzed. Tests against the scale mean of four reveal that the robot's behavior was generally evaluated as intentional [*M* = 4.73, *t*_(10)_ = 2.39, *p* = 0.019, *d* = 4.69], surprising [*M* = 5.18, *t*_(10)_ = 2.55, *p* < 0.014, *d* = 3.37], and desirable [*M* = 4.64, *t*_(10)_ = 1.41, *p* = 0.095, *d* = 3.09]. Ratings with regard to the robot's behaviors' understandability were not statistically different from 4. Further, the robot was perceived as likable [*M* = 5.24, *t*_(10)_ = 4.84, *p* < 0.001, *d* = 6.18], trustworthy [*M* = 4.58, *t*_(10)_ = 4.58, *p* = 0.004, *d* = 7.86] and intelligent [*M* = 4.89, *t*_(10)_ = 4.36, *p* < 0.001, *d* = 7.220]. As a response to the question which moments participants perceived as natural during the interaction, seven participants referred to the conversation flow and four explicitly named the nonverbal behavior, suggesting that the multimodal behavior generation was overall perceived as naturalistic. When asked about unnatural behavior, five participants referred to the verbal interaction being not entirely intuitive, referring to difficulties with regard to turn-taking and seven participants referred to the robot's stretching or moving away without previous notice as irritating.

#### 5.2.2. Self-Explanation Generation

The explainability requirements that refer to individual architecture components (Requirement 1) and their communication interfaces (Requirement 2) could not be explicitly tested with the interaction, but are indirectly validated *via* assessment of Requirements 3–5. The interaction protocols reveal that 7 *what-*, 8 *why-*, and 1 *elaboration-* request were correctly answered. This assesses the robot's capability to understand verbal requests from the user, to dynamically select the correct explanation type, and to retrieve the information needed for an explanation from within the architecture (inspectability). The robot was thus, in general, able to deliver user-centered explanations (Requirement 5), further validating fulfillment of Requirements 1–4.

Strikingly, out of the 16 correctly identified requests, nine were uttered while no action (5) or strategy (4) was currently active, leading to participants receiving a correct, but only sparsely helpful answer (“I am currently doing nothing/not pursuing any goal”). While this can be attributed to the explanation info being updated incrementally and in parallel to the behavior generation, and attests fulfillment of Requirement 4, the outcome is not desirable and should be incorporated in the explanation dialog flow model. For instance, in this specific case, one could switch to past episodes to explain the last active action/strategy if no active strategy is currently available. This observation is in line with the survey data: while the robot's explanations were rated as understandable [mean statistically significantly higher than scale mean of 4: *M* = 5.27, *t*_(10)_ = 3.55, *p* = 0.003, *d* = 4.43], the explanations were not rated as particularly justifying the robot's behaviors (*M* = 4.09) or helping participants understand why the robot behaved as it did (*M* = 4.0).

Interestingly, further inspection of the protocols reveals that these explanation requests were often uttered in relation to the robot's action of driving away from the user. This behavior was reactive, being directly triggered *via* an action command sent from the dialog manager and thus not represented in the robot's self-model for explanation generation. While this is a shortcoming of the current implementation, it simultaneously supports the underlying claim that the robot's behavior needs to be explainable at any time. Notwithstanding, the seven explanations uttered while a strategy or behavior was currently being executed show that the robot was able to successfully verbalize the current behavior with the adequate explanation strategy if deliberately chosen *via* its internal decision process.

In addition, while the above examples demonstrate that the robot is in principle able to provide behavioral self-explanations, a large number of verbal explanation requests by the user were not correctly identified (7 what, 3 why, and 3 elaboration requests) and were thus not answered correctly. Improvement of the processing of language-based explanation request thus proves essential: In addition to the expansion of NLU training data, it seems indispensable to consider the socio-interactive context when processing the user's verbal input. This goes in hand with participants' broad consensus regarding suggestions for improvement: 9 of 11 participants mentioned insecurities with respect to the verbal interaction with the robot. Participants reported issues such as non-intuitive turn-taking due to the robot's response times and feeling unsure about conversational roles especially mentioning switches between system-initiated dialog sequences (for example during *StrategyAcquaintWithUser*) and sequences where the robot did not have the dialog initiative. These difficulties with the dialog management, the relatively large number of “no operation” explanations and mis-recognized requests may also offer an explanation for the overall relatively small number of elaboration requests.

## 6. Conclusion

In this article, we have presented work toward social robots capable of giving (self-)explanations of their own needs-based behavior, at any time during a running interaction. These explanations are socio-interactively constructed to effectively fit the current information need of a human user. To that end we have combined insights from research on human-robot interaction architectures as well as explanation generation, in order to derive concrete requirements for explainable behavior generation architectures of social robots. We have formulated a socio-interactive framework for self-explanation generation, incorporating empirically validated explanation strategies and closely embedded in the behavior generation architecture. Further, we have described an implementation of this architecture realizing this concept and addressing the requirements.

A first evaluation has proven that the proposed architecture presents a useful foundation to meet the challenges of explainable autonomous behavior generation. As previously stated, the results of this initial experiment were gathered with a confined user group interacting with the robot in a first basic greeting use case. Therefore, applicability to divergent target groups and transferability to more complex use cases need to be assessed in future experiments. Nevertheless, our results suggest that the socio-interactive approach is, first, conceptually and technically feasible and, second, accepted and appreciated by real users: participants ask what the robot is doing and why it is doing so, and the robot is able to introspect its reasoning and to present it in ways that were previously shown to enhance user understanding and acceptance of the robot's behavior—necessary pre-requisites to support user-centered explanation delivery. At the same time, our evaluation has provided valuable insights on how to improve the technical realization: while the robot is able to correctly address users' explanation requests in specific situations (behavior is currently being executed, request is correctly identified, etc.), some explanation requests were not adequately answered by the robot (e.g., concerning reactive behavior). This showcases the necessity to increase the level of socio-interactivity by linking perception with explanation generation.

Accordingly, we identify the following starting points to extend the current implementation: in order to improve the processing of users' explanation requests, the NLU needs to be improved by extending its underlying training data, and also by grounding it more strongly in the interaction context. Similarly, the robot's explanation capabilities need to be extended such that the robot, in addition to its deliberately chosen behaviors, is also able to introspect its reactive nonverbal and verbal behaviors that were executed as a direct response to events in the environment. This can be enabled by extending the robot's self model and episodic memory to link all behaviors to the (internal or external) events in their causal history. This leads to another important extension: giving memory-based explanations for past behavior, for example if the robot is currently doing nothing, but executed an action shortly before the user's explanation request. For this, the recently closed interaction episode could be checked. Taking this one step further, explanations could entail causal links that express events that led to changes of robot needs, which in turn led to selection of a certain strategy (akin to Malle, 1999's) causal history of reasons). These explanation types would need to be empirically validated before implementation in the architecture in order to fulfill Requirement 3.

Similarly, on the behavior generation level, future work needs to address the robot's capabilities to perceive its surroundings and dynamically incorporate changes in the environment in its behavioral decisions. This includes, but is not limited to, reliably perceiving visual and auditory stimuli, and linking them to memory events such as a user being present and, eventually, the user's current activity status or information need. In the long term, behavior selection and execution, as well as explanation delivery should be adapted to each user individually. Users' reactions to the robot's behavior as well as timing and type of explanation requests could be used to learn users' preferences and shape the robot's behavior planning process (Umbrico et al., 2020). This would lead to differences in the strategy selection or parameterization of specific actions, considering user traits such as preferences regarding proxemics. This could increase the social interaction's naturalness by balancing the robot and the user's needs more harmonically. Eventually, this should lead to a form of “proactive explainability” of the social robot, in that it may actively offer explanations to the user, may learn to predict which of its behavior requires explanations, or may even consider user-centered estimates of interpretability or explainability in its own behavioral choices. While aiming at the best possible adaptation of behavioral and explanatory decisions to the users' preferences, the explainability requirements should persist as a crucial criterion to drive further development: learning approaches should be incorporated meaningfully, without compromising inspectability of the robot's reasoning.

Finally, we underscore the importance of embedding explanations in a larger framework that accounts for the socio-interactive mechanisms with which understanding is achieved (explaining being one of them). The proposed architecture has proven to be a useful approach to explainable social behavior generation. At the same time the evaluation has shown that, in order to exploit the full potential of such an integrated approach, the socio-interactive capabilities of robots have to be extended further, enabling more robust perception of the users' needs for explanation and thus enabling social robots to optimally deploy self-explanations, increasing the understandability and desirability of robot behavior for their human users.

## Data Availability Statement

The raw data supporting the conclusions of this article will be made available by the authors, without undue reservation.

## Ethics Statement

The studies involving human participants were reviewed and approved by Ethik-Kommission der Universität Bielefeld (EUB). The patients/participants provided their written informed consent to participate in this study. Written informed consent was obtained from the individual(s) for the publication of any potentially identifiable images or data included in this article.

## Author Contributions

SS and SK conceived the explanation dialog framework. TH, SK, and SS devised the explainable behavior architecture. TH, FS, SS, and JK implemented and tested the architecture, wrote sections of a draft manuscript, and designed the figures. SS, FS, JK, and SK designed the study. SS administered and prepared the study and performed statistical analysis of the questionnaire data. FS, JK, and SS conducted the study and analyzed the qualitative interaction data. SS, TH, and SK revised and finalised the manuscript after review. All authors contributed to the manuscript, read and approved it for submission.

## Funding

This research was supported by the German Federal Ministry of Education and Research (BMBF) in the project VIVA (FKZ 16SV7959). This research was also partially supported by DFG in the SFB/TRR 318 Constructing Explainability. We acknowledge support for the publication costs by the Open Access Publication Fund of Bielefeld University and the Deutsche Forschungsgemeinschaft (DFG).

## Conflict of Interest

The authors declare that the research was conducted in the absence of any commercial or financial relationships that could be construed as a potential conflict of interest.

## Publisher's Note

All claims expressed in this article are solely those of the authors and do not necessarily represent those of their affiliated organizations, or those of the publisher, the editors and the reviewers. Any product that may be evaluated in this article, or claim that may be made by its manufacturer, is not guaranteed or endorsed by the publisher.

## Footnotes

^1^ https://navelrobotics.com/viva/

^2^ http://dlib.net/

^3^ http://blog.dlib.net/2017/02/high-quality-face-recognition-with-deep.html

^4^ http://www.iis.fraunhofer.de/shore

^5^ https://cloud.google.com/speech-to-text

^6^ https://rasa.com/docs/rasa/nlu-only/

^7^ https://spacy.io/models/de

^8^All vectors are column vectors.

^9^More elaborate strategies to handle conflicting needs (e.g., suppression, deference, etc.) would be explored in the future.

^10^ https://py-trees.readthedocs.io/

^11^ https://www.softbankrobotics.com/emea/en/pepper

^12^ https://developer.softbankrobotics.com/pepper-naoqi-25

^13^ https://gitlab.ub.uni-bielefeld.de/scs/ipaaca

^14^ https://youtu.be/yHl-hTbRECQ

^15^ https://www.soscisurvey.de/

^16^ https://jasp-stats.org/
